# A high dose of conjugated linoleic acid increases fatty liver and insulin resistance in lactating mice

**DOI:** 10.1371/journal.pone.0214903

**Published:** 2019-08-07

**Authors:** Kun Pang, Zhongke Zhu, Songbo Zhu, Liqiang Han

**Affiliations:** 1 College of Animal Science and Veterinary Medicine, Xinyang Agriculture and Forestry University, Xinyang, China; 2 Zhengzhou Academy of Agriculture and Forestry Sciences, Zhengzhou, China; 3 College of Animal Science and Veterinary Medicine, Henan Agricultural University, Zhengzhou, China; Max Delbruck Centrum fur Molekulare Medizin Berlin Buch, GERMANY

## Abstract

This study aimed to evaluate the effects of a high dose of conjugated linoleic acid (CLA) on lactating mice. In one experiment, Kunming mice were separated into four groups (n = 6 per group); the control (CON) group received 3.0% linoleic acid (LA) oil, the L-CLA group received a mixture of 2.0% LA and 1.0% CLA, the M-CLA group received a mixture of 1.0% LA and a 2.0% CLA, and the H-CLA group received 3.0% CLA. Feeding proceeded from day 4 to day 10 of lactation. In a second experiment, a CON group received 3.0% LA, and an H-CLA group received 3.0% CLA. Plasma parameters were analyzed for all groups, and insulin tolerance tests (ITTs) were conducted. CLA treatment did not affect dam weight but significantly decreased the food intake of dams during lactation. Furthermore, CLA decreased the weight of pups on day 10 of lactation; this effect was attributed to lower milk fat of dams in the CLA group than in those of the other groups. Relative to mice in the CON group, the mice in the H-CLA group displayed increased liver weight and liver triglyceride (TG) content as well as higher TG content and γ-glutamyl transferase (γ-GT) activity in the plasma. Moreover, high-dose CLA resulted in insulin resistance, possibly affecting the red blood cell (RBC) and hemoglobin (HCB) levels in the plasma. In conclusion, lactating mice receiving a high dose of CLA exhibited fatty liver, insulin resistance, and impaired lactation performance.

## Introduction

Conjugated linoleic acids (CLAs) are molecules mostly found in meat and dairy products. CLAs have emerged as a possible adjuvant treatment for obesity because some studies show a reduction in body fat and increased lean mass after supplementation of a mixture of two CLA isomers in the diet [1,2]. In addition, the oil of CLAs is reported to affect carcinogenesis, glucose and lipid metabolism, diabetes, body composition, and immune cell functions [3–5]. However, certain isomers seem to cause fat accumulation in enlarged livers of mice [6] and can induce insulin resistance in mice and humans [7,8].

In humans and other animals, milk lipids are markedly affected by the lipid composition of the diet, maternal energy balance, and maternal metabolism. Maternal lipid intake during lactation can alter milk composition, which in turn can affect the growth of the litter [9] and change lipid metabolism and liver enzymes activities [10]. Many studies have shown that CLAs can lower triacylglycerol concentrations and alter the fatty acid profile of the milk of lactating animals, including humans, mice, rats, sheep, goats and cows [11–17]. Feeding lactating mice with CLA can result in diminished mammary gland lipogenesis and reduced growth rates [18,19], but the exact metabolic and physiological changes involved remain unclear. In addition, the intake of trans-fatty acids during lactation has been found to increase maternal lipids in the liver [13] and liver weight [20].

Recently, Bezan et al (2018) demonstrated that feeding male Wistar rats a high dose (3%) of CLA, including an equimolar mixture of c9,t11-CLA and t10,c-12 CLA, increased insulin resistance and hepatic triacylglycerol accumulation in the rats [21]. We hypothesized that high doses of CLA administered to lactating mice may cause liver damage and insulin resistance. To test this hypothesis, two experiments were performed in which mice were fed CLA mixtures. In the first experiment, the effects of different doses of CLA on the milk and liver of dams were investigated. In the second experiment, we tested whether a high dose of CLA could cause insulin resistance in dams.

## Materials and methods

### Animals and diets

This study was carried out in strict accordance with the recommendations in the Guide for Henan Laboratory Animal Center for Medical Sciences Animal Care and Use Committee. The protocol was approved by the Committee on the Ethics of Animal Experiments of Henan Agricultural University (Protocol Number: 18–102). Kunming (KM) mice were used in this study and are the most productive and outbred mice in China. They are derived from Swiss mice. KM mice were housed at 24°C under 40% humidity and a 12/12 h light-dark cycle. Animals were provided with food and water ad libitum. A basal mouse diet (prepared by Henan Laboratory Animal Central) containing the required complement of nutrients for lactation was used as a carrier for each of the four fatty acid treatments. The basal diet was prepared by mixing the powdered ingredients in the following concentrations (g/kg diet): casein, 235; soybean oil, 50; maize starch, 438; sucrose, 193; AIN-93 mineral mix, 35; AIN-93 vitamin mix, 10; cellulose, 33; choline bitartrate, 3; and DL-methionine, 3. The oil of LA (linoleic acid) and CLA was purchased from Zhongshan Unicare Natural Medicine Co., Ltd. The fatty acid compositions of the CLA and LA oils are shown in Table 1. The diets were prepared by the Henan Laboratory Animal Center by adding the LA oil and/or CLA oil by weight to the basic powder diet and mixing the ingredients with water for dissolution. The diets were prepared the day before parturition and stored at 4°C.

**Table 1:** Compositions of LA oil and CLA oil.

Composition	CLA (%)^a^	LA (%)
Palmitic acid (C16:0)	4.89	3.58
Stearic acid (C18:0)	2.15	1.26
Oleic acid (C18:1)	10.64	11.21
Linoleic acid (C18:2)	0.75	80.13
Conjugated linoleic acid (C18:2)	81.48	-
c9,t11 CLA	37.53	-
t10,c12 CLA	38.38	-
Other CLAs	5.57	-
Linolenic acid (C18:3)	0	2.35
Other fatty acids	0.09	1.47

^a^ CLA, conjugated linoleic acid; LA, linoleic acid

### Experiment one

In experiment one, all dams were fed the laboratory basal diet for the first 3 days of lactation. On day 4 postpartum, six lactating mice per group were randomly assigned to a control group (CON group, 3.0% LA oil), a low-level CLA group (L-CLA group, 1.0% CLA + 2.0% LA), a medium-level CLA group (M-CLA group, 2.0% CLA + 1.0% LA), and a high-level CLA group (H-CLA group, 3.0% CLA) and treated from day 4 to day 10 postpartum. The litter number was normalized to eight on the day of birth. Dam weight, food intake, and the litter weight of a total of eight pups were determined daily between days 4 to 10 postpartum, as described in detail previously [22]. Residual feed intake was measured at the end of each day to determine daily feed intake, taking into account spilled feed. Based on the previous day’s intake, a sufficient amount of feed was offered to ensure the diets were available ad libitum. On day 10 postpartum, pups were separated from dams for 2 h before feeding to ensure the maximal accumulation of milk in the mammary gland. Subsequently, dams and pups were placed together to let the pups nurse and obtain adequate milk and to allow time for milk clots to form in the stomach. Then, the pups were euthanized by cervical dislocation. Their abdomens were opened, and milk clots within the stomach were weighed and stored at -78°C until analysis [12,23]. Dams were euthanized by cervical dislocation on day 10 of lactation. Mammary and liver tissue from dams were excised and weighed, frozen in liquid nitrogen, and stored at -80°C.

#### Analysis of milk and liver samples

The milk lipid concentration was measured gravimetrically after chloroform-methanol extraction by a modified Folch method [24]. Protein concentration was determined by the Biuret method with casein as the standard [25]. The milk lactose concentration was estimated by the Cocciardi method [26]. A total of 50 mg liver tissue was homogenized by adding 1 mL pyrolysis solution. The mixture was left to stand for 10 min, and then the supernatant was transferred to a 1.5 mL centrifuge tube, heated for 10 min at 70°C and centrifuged for 5 min at room temperature at 2,500 ×g. The triglycerides of the supernatant were analyzed using a Tissue Triglyceride Assay Kit (E1013, Applygen Technologies Inc.) according to the manufacturer’s protocol. The intra- and interassay coefficients of variation were 3.5 and 3.7%, respectively, for milk fat, 3.5 and 4.0%, respectively, for milk protein, 2.6 and 3.4%, respectively, for milk lactose and 4.8 and 4.1%, respectively, for liver TG.

### Experiment two

In experiment two, dams received 3.0% LA oil (CON group) or 3.0% CLA oil (H-CLA group) from day 4 to day 10 of lactation. The feeding conditions were the same as in the first experiment. On the 10th day of lactation, whole blood was collected from the dam’s tail vein with a 1.5 mL centrifuge tube containing heparin, and plasma was obtained by centrifugation at × g for 20 min at 4°C.

#### Blood parameter analysis

The white blood cell (WBC), red blood cell (RBC), platelet (PLT) and hemoglobin (HGB) levels were analyzed by a blood cell analyzer (Coulter LH780). The plasma metabolite concentrations of cholesterol (CHO), triglyceride (TG), low-density lipoprotein (LDL), high-density lipoprotein (HDL), glucose, alanine aminotransferase (ALT), aspartate aminotransferase (AST), γ-glutamyl transferase (γ-GT) and alkaline phosphatase (ALP) were analyzed by a fully automatic chemistry analyzer (Cobas P8000, Roche) at the Hospital of Zhengzhou University. Plasma insulin concentrations were quantified using a Mouse Insulin Ultrasensitive ELISA Kit (BC1710, Solarbio). The index of insulin resistance (HOMA-IR) was calculated with the formula Fasting blood glucose (mmol/L)×Fasting Insulin (μIU/mL)/22.5.

#### Insulin tolerance test

For the insulin tolerance test (ITT), on the 11^th^ day of lactation, all pads were renewed to prevent mice from eating food that had spilled on the pad. After being fasted for 6 h, each dam was weighed and intraperitoneally injected with insulin (100 units/mL, Novolin R, Novo Nordisk, Denmark) at a dose of 0.75 IU/kg body weight. Before injection, the baseline blood glucose concentration was measured and recorded as the concentration at 0 min. After insulin injection, the blood glucose level in the tail vein was measured at 15, 30, 60, 90 and 120 min using a TIDEX glucose analyzer (Sankyo, Tokyo). The rate constant for each ITT (KITT) was calculated using the equation KITT (%/min) = 0.693 / t(1/2), where t(1/2) was calculated from the slope of the plasma glucose concentration 15–120 min after the administration of intravenous insulin.

#### Statistical analysis

The results are reported as the mean ± standard deviation (SD). Descriptive statistics were calculated and analyses of differences among groups were analyzed by SPSS 13.0 (IBM, USA). All experiments were conducted in triplicate. The data for dam body weight, food intake and total body weight of eight pups in experiment one and ITT in experiment two were analyzed for significant differences among or between groups using a general linear model for analysis of variance with the factors of diet (D), time (T) and their interaction (D×T). The effects of CLA on the milk and liver of dams in experiment one were analyzed using a one-way analysis of variance (ANOVA). Tukey’s test was used for multiple comparisons. The plasma parameters in experiment two were analyzed using Student’s t-tests. A *P*-value <0.05 was considered statistically significant.

## Results

### Experiment one

#### Dam body weight and food intake

**Fig 1:**
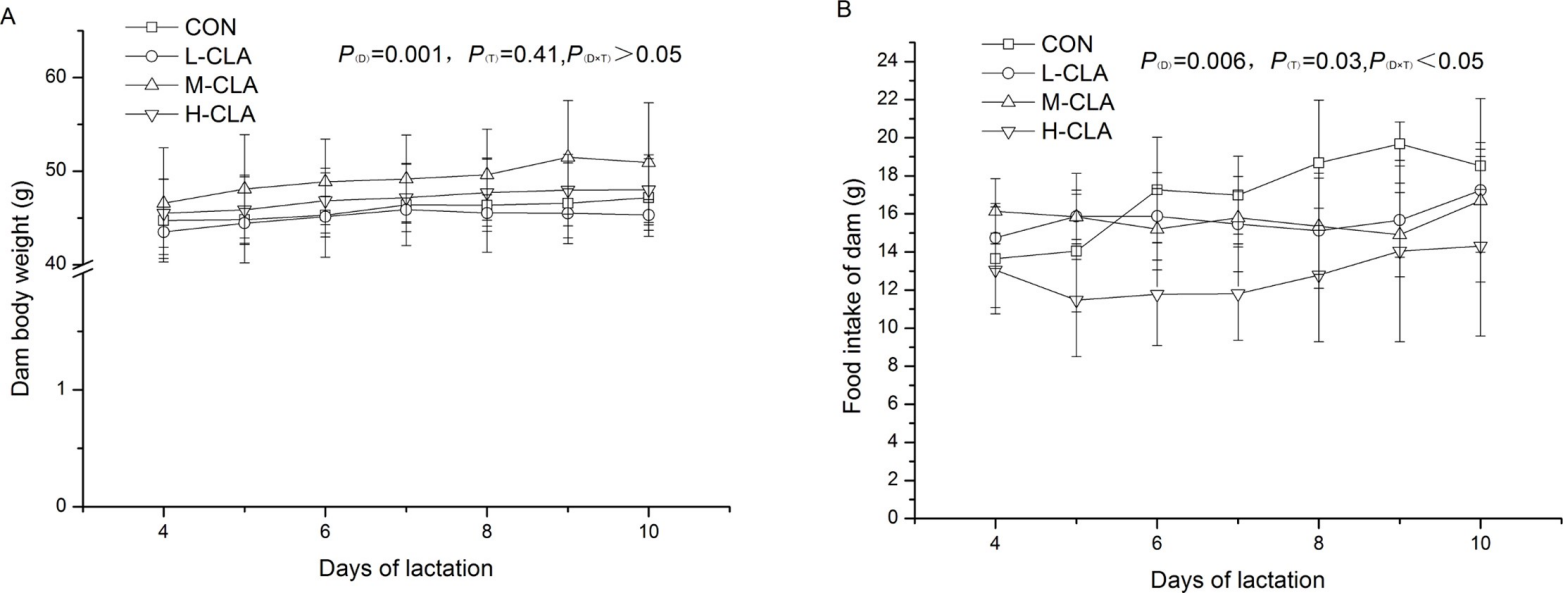
Daily body weight and food intake of dams fed different doses of CLA. Values are means ± SDs (n = 6). (A) Daily body weight of dams. (B) Food intake of dams.CON group, 3.0% LA oil; L-CLA group, 1.0% CLA + 2.0% LA; M-CLA group, 2.0% CLA + 1.0% LA; H-CLA group, 3.0% CLA; D, effect of lactation time; T, effect of CLA treatment; D×T, time and treatment interaction.

In experiment one, dam body weight increased linearly over the course of the experiment, with a significant time effect (*P* <0.01, Fig 1A), but there were no significant differences among the treatment groups. Significant effects of treatment and time were observed for food intake of dams (*P* <0.05, Fig 1B). The food intake of dams in the control group was significantly higher than the food intakes of dams in the CLA groups.

#### Pup body weight

**Fig 2:**
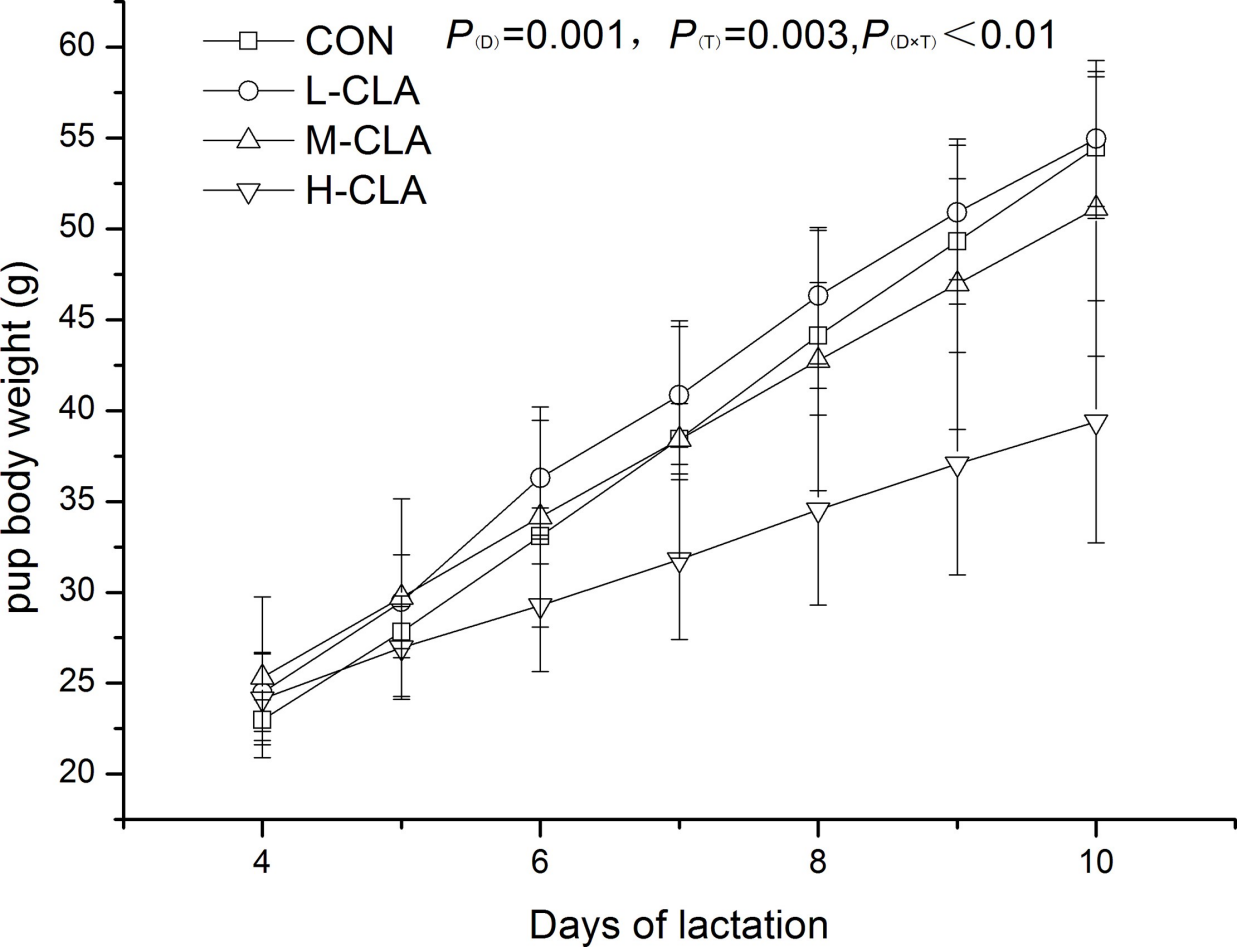
Daily weight of nursing pups fed different doses of CLA from day 4 to day 10 of lactation. Values are means ± SDs (n = 6). CON group, 3.0% LA oil; L-CLA group, 1.0% CLA + 2.0% LA; M-CLA group, 2.0% CLA + 1.0% LA; H-CLA group, 3.0% CLA; D, effect of lactation time; T, effect of CLA treatment; D×T, time and treatment interaction.

The total weight of eight pups in experiment one is shown in Fig 2. Significant effects of both time and treatment were observed on total pup weight gain over the feeding period. Relative to the control treatment, CLA treatment significantly reduced the total body weight of pups. The total eight-pup weights in the H-CLA group were significantly lower than those in the M-CLA, CON and L-CLA groups (39.38 g vs. 51.13 g, 54.47 g, and 54.95 g, respectively, *P* <0.01) by day 10 of lactation.

#### Milk and liver of dams

The milk clot weight obtained from the stomach ranged between 0.91 and 0.56 g/suckling pup. The milk fat content of the H-CLA group was significantly lower than the contents of the other groups. There were no significant differences in protein and lactose concentrations in milk among the CLA and control groups (Table 2). Similarly, there was no difference in the weight of mammary tissue among the groups. Liver weight increased gradually with increasing dose of CLA, and there was a significantly higher TG content in the liver in the H-CLA group than in the other groups (Table 2).

**Table 2:** Effects of CLA on the milk and liver of dams.

	CON ^#^	L-CLA	M-CLA	H-CLA	*P*-value
Milk clot weight (g/pup)	0.89 ± 0.27 ^a^	0.91 ± 0.12 ^a^	0.85 ± 0.23 ^a^	0.56 ± 0.29 ^b^	0.018
Milk fat (mg/100 mg)	15.42 ± 1.43 ^a^	14.16 ± 1.96 ^a^	13.37 ± 2.30 ^a^	11.88 ± 1.99 ^b^	0.004
Milk protein (mg/100 mg)	18.79 ± 1.35	20.19 ± 2.30	17.96 ± 3.71	19.87 ± 3.32	0.296
Milk lactose (mg/100 mg)	2.03 ± 0.56	1.89 ± 1.45	2.26 ± 0.85	1.93 ± 1.30	0.889
Mammary weight (g)	3.88 ± 0.69	4.21 ± 0.79	4.39 ± 1.23	3.96 ± 1.06	0.09
Liver weight (g)	2.91 ± 0.29 ^a^	3.02 ± 0.41 ^a^	3.63 ± 0.55 ^a^^b^	4.65 ± 0.59 ^b^	0.02
TG of liver (mg/g tissue)	46.41 ± 27.07 ^a^	94.51 ± 32.78 ^a^^b^	92.5 ± 34.16 ^a^^b^	230.14 ± 80.54 ^b^	<0.001
Liver weight/body weight (%)*	6.29±0.93	6.63±0.85	7.37±0.82	7.56±1.44	0.11

^a,b^Values in the same row followed by different letters are significantly different (*P* <0.05). Values are means ± SDs.

^#^CON group, 3.0% LA oil; L-CLA group, 1.0% CLA + 2.0% LA; M-CLA group, 2.0% CLA + 1.0% LA; H-CLA group, 3.0% CLA

* weight of liver/weight of dam body×100%

### Experiment two

#### Body weight of pups

**Fig 3:**
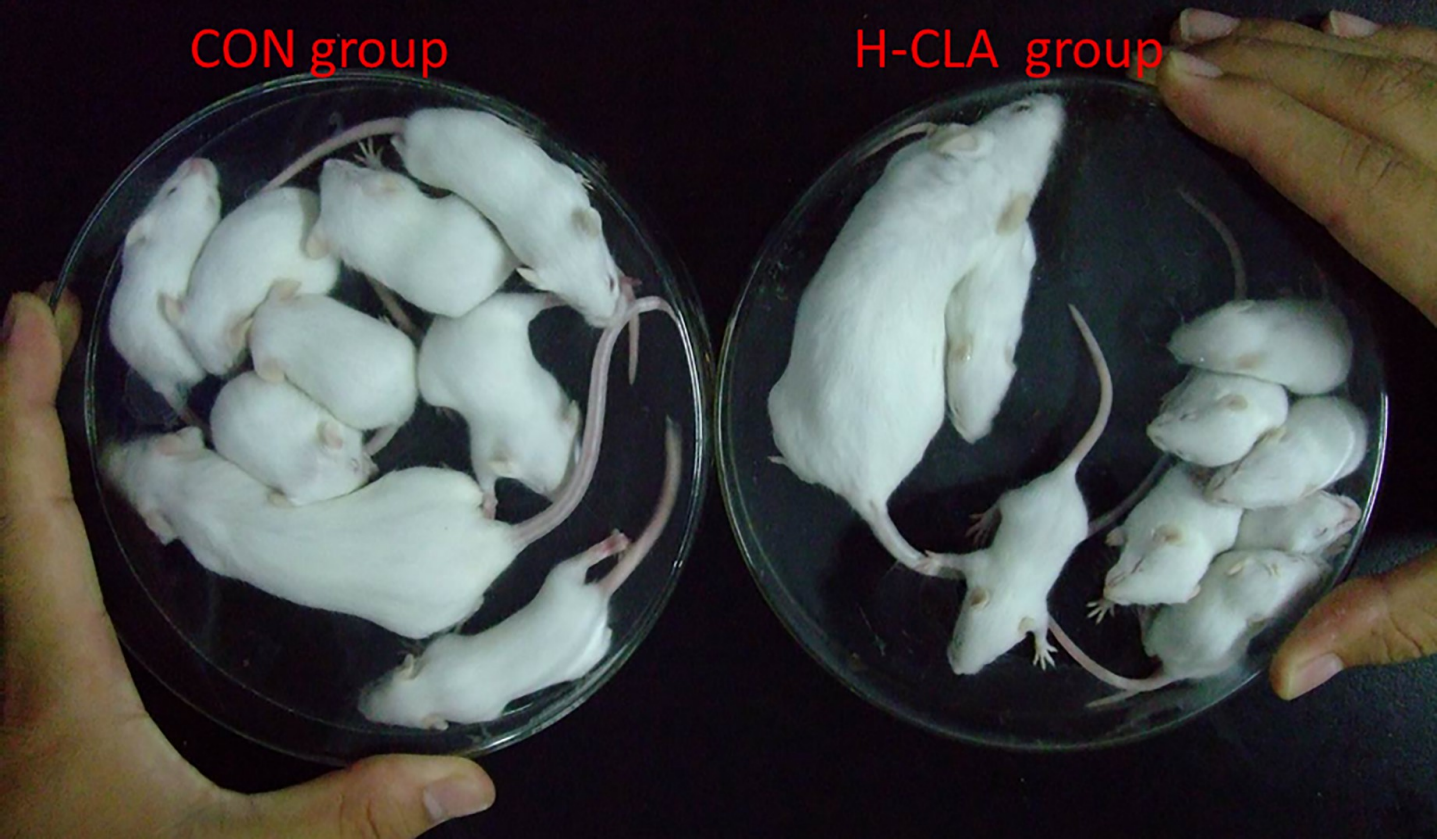
Dams and eight pups of the CON and H-CLA groups at day 10 of lactation.

Similar to pup weight in experiment one, in experiment two, the weight of the pups in the high-dose CLA group was significantly lower than that in the control group (Fig 3).

#### Blood metabolite analysis

CHO, HDL and LDL levels in the serum were unaffected by treatment. However, plasma TG and γ-GT activity were higher in the H-CLA treatment group than in the control group (*P* <0.05), as was glucose (*P* = 0.07, Table 3). Insulin activity and HOMA-IR were significantly higher in the H-CLA group than in the control group (*P* <0.001). Interestingly, compared with feeding in the control group, CLA feeding resulted in higher RBC (8.66×10^12^/L vs. 7.35×10^12^/L, *P* <0.01) and HGB (140 g/L vs. 117 g/L, *P* <0.01).

**Table 3:** Effect of high-dose CLA on the blood metabolites of dams.

Parameter^a^	CON group^b^	H-CLA group	*P*-value
CHO (mmol/L)	2.52 ± 0.38	2.86 ± 1.01	0.461
TG (mmol/L)	1.04 ± 0.34	2.17 ± 1.26	0.044
HDL (mmol/L)	1.53 ± 0.21	1.56 ± 0.46	0.887
LDL (mmol/L)	0.22 ± 0.08	0.29 ± 0.05	0.162
Glucose (mmol/L)	6.58 ± 1.20	7.49 ± 1.53	0.070
ALT (U/L)	39.20 ± 10.51	41.00 ± 27.38	0.896
AST (U/L)	69.40 ± 13.97	74.16 ± 7.28	0.560
γ-GT (U/L)	15.40 ± 3.92	24.50 ± 2.98	0.005
ALP (U/L)	22.00 ± 8.02	28.50 ± 2.29	0.183
Insulin (μIU/mL)	6.38 ± 1.72	27.15 ± 4.91	<0.001
HOMA-IR	1.90 ± 0.59	8.92 ± 4.73	<0.001
WBC (10^9^/L)	3.31 ± 1.74	2.94 ± 1.28	0.734
RBC (10^12^/L)	7.35 ± 0.47	8.66 ± 0.34	0.002
PLT (10^9^/L)	1415.83 ± 84.93	1326.00 ± 196.74	0.431
HGB (g/L)	117.00 ± 8.10	140.00 ± 4.04	0.002

^a^CHO, cholesterol; TG, triglyceride; LDL, low-density lipoprotein; HDL, high-density lipoprotein; ALT, alanine aminotransferase; AST, aspartate aminotransferase; γ-GT, γ-glutamyl transferase; ALP, alkaline phosphatase; WBC, white blood cell; RBC, red blood cell; PLT, platelet; HGB, hemoglobin

^b^CON group, 3.0% LA oil; H-CLA group, 3.0% CLA oil

#### Insulin tolerance test

**Fig 4:**
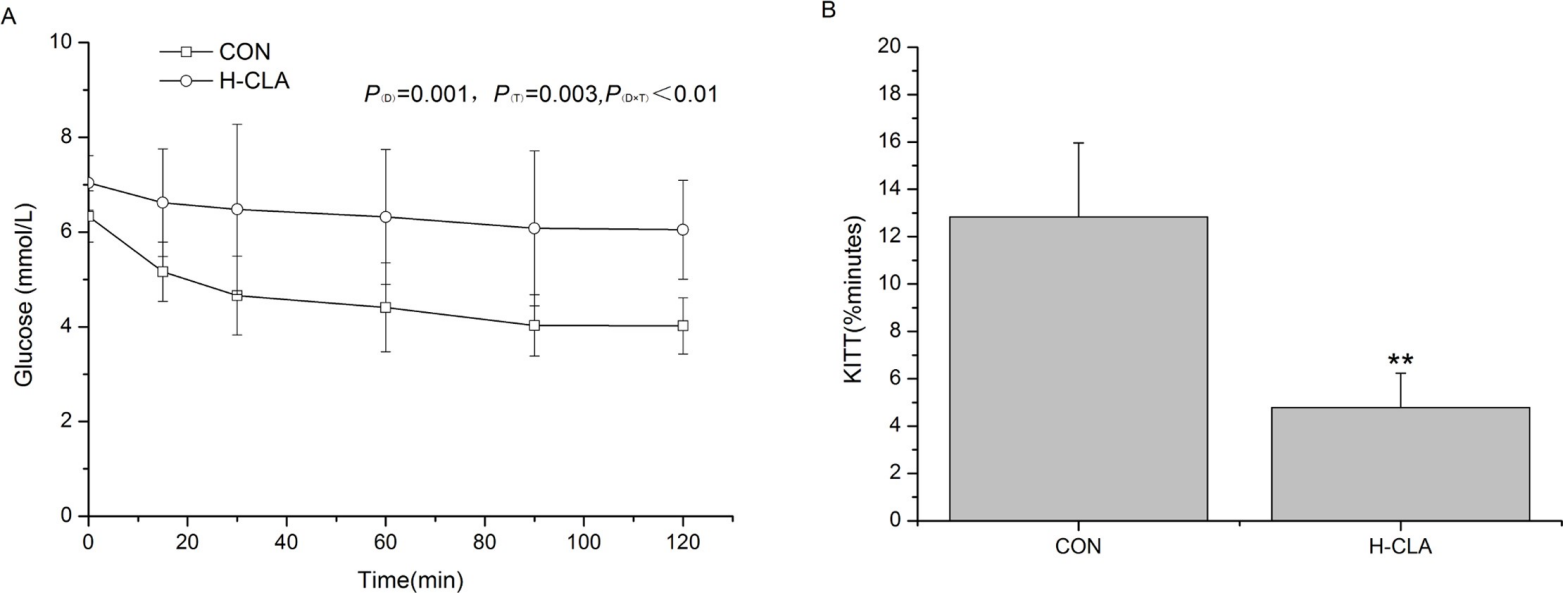
Insulin tolerance test results of lactating mice in the CON and H-CLA groups. Values are means ± SDs (n = 6). (A) ITT values of lactating mice. (B) Calculation of KITT (%min) after a dose of 0.75 U of insulin per kg of body weight. CON group, 3.0% LA oil; H-CLA group, 3.0% CLA; ITT: insulin tolerance test; KITT: rate constant for ITT; **P <0.01.

Significant effects of treatment and time on ITT were observed (*P* <0.01, Fig 4A). At 0 min, the blood glucose level in the control group was 6.62 mmol/L, and that in the CLA group was 7.04 mmol/L (*P* > 0.05, Fig 4A). After insulin injection, glucose in the control group decreased to 4.02 mmol/L at 120 min, whereas glucose levels in the H-CLA group did not markedly decrease (6.08 mmol/L). In addition, the rate constant of ITT in the H-CLA group was significantly lower than that in the control group (*P*<0.01, Fig 4B).

## Discussion

CLA can reduce fat deposition, weight gain, and food/energy intake in mice [27]. In the present study, CLA treatment decreased food intake in lactating dams. Increased maternal intake of CLA during lactation has been shown to enhance the growth rate of suckling neonatal rats [28]. However, in contrast to observations of the stimulatory effects of CLA, mixtures of CLA [19] or isoforms [18] have been found to cause litter pups to grow slowly or even die due to decreased milk fat. These contradictory results might be due to study differences in the content or isomeric distribution of the CLA supplement. In our present study, a 3% CLA diet fed to KM mice during lactation decreased both milk clot weight and milk fat content. Milk is the only source of nutrients for suckling pups; hence, it was not surprising that the reductions in the fat and weight of milk were accompanied by reduced pup weight in the pups of lactating dams. These findings confirm that a high dose of CLA can lower the content of milk fat and pup growth rate. Although the milk FA composition was not analyzed in the present study, Harvatine et al (2014) found that CLA treatment decreased the concentration of de novo synthesized FAs (12:0 and 16:0) and increased the concentration of PUFA in milk fat (20:4n–6 and 22:4n–6), which suggest that CLA treatment decreased the lipogenic capacity of mammary tissue [12].

CLA has been reported to generate fatty liver, which could be a consequence of increased lipogenesis in the liver to compensate for the reduction in fat deposition in adipose tissue [27]. We assessed the liver of dams and observed increased liver weight and TG content in mice given a high dose of CLA. Kadegowda et al (2010) reported that mice treated with CLA displayed decreased milk fat and increased liver weight due to hepatic steatosis [29], which is consistent with our current results. The effects of CLA supplementation on mammary fat synthesis have received a great deal of attention [30,31], but the potential adverse effects of CLA on the liver and lipid metabolism in lactating animals have been largely ignored. We suggest that severe lipid accumulation occurs in the liver when lactating mice are given high doses of CLA. This suggestion is supported by the increased TG content and γ-GT activity in the serum of mice in the high-CLA group relative to CON group.

There have been many reports regarding CLA-induced insulin resistance [7]. In the present study, the HOMA-IR and ITT results indicated that the high dose of CLA fed to the mice caused insulin resistance. In lactating mice, glucose is the main precursor for the synthesis of lactose and triglyceride substrates. During the lactation stage, mammary glands have increased demand for glucose and are highly sensitive to insulin, whereas other organs are relatively insensitive to insulin [32]. In this study, lactating mice fed CLA displayed aggravated insensitivity to insulin and exhibited insulin resistance, implicating insulin as a key factor in CLA-mediated regulation of milk fat and lactation.

Interestingly, we observed for the first time that a high dose of CLA can result in higher RBC and HGB levels in the whole blood of lactating mice. RBC and HGB are both positively correlated with insulin resistance and nonalcoholic fatty liver disease (NAFLD) [33]. Thus, in the present study, the higher RBC and HGB levels in the mice receiving the high dose of CLA may have been consequences of insulin resistance and fatty liver.

In summary, a high level of CLA ingestion during lactation decreases milk fat and increases lipid content in the liver. These findings indicate that dietary CLA has the propensity to affect hepatic metabolism and lactation. Little is known about the complex regulatory mechanism between lipid metabolism in the liver and mammary lactation. The present findings provide a platform for further studies on this mechanism and the safe use of CLA.
